# Is there an association between maternal anxiety propensity and pregnancy outcomes?

**DOI:** 10.1186/s12884-018-1925-8

**Published:** 2018-07-04

**Authors:** Eyal Ravid, Liat Salzer, Liat Arnon, Michal Eisner, Arnon Wiznitzer, Aron Weller, Lee Koren, Eran Hadar

**Affiliations:** 1 Sackler Faculty of Medicine, Tel Aviv University, Tel Aviv, Israel; 2 Helen Schneider Hospital for Women, Rabin Medical Center, 49100 Petach-Tikva, Israel; 3 Mina and Everard Goodman Faculty of Life Sciences, Bar-Ilan University, Ramat-Gan, Israel; 4 Department of Psychology and the Gonda Brain Research Center, Bar-Ilan University, Ramat-Gan, Israel

**Keywords:** Anxiety, Pregnancy, Adverse outcome

## Abstract

**Background:**

Several studies have shown inconsistent associations between anxiety during pregnancy and adverse pregnancy outcome. This inconsistency may be due to lack of controlling for the timing and type of maternal anxiety. We aimed to isolate a specific type of anxiety - maternal anxiety propensity, which is not directly related to pregnancy, and evaluate its association with adverse pregnancy outcome.

**Methods:**

We conducted a prospective observational study of 512 pregnant women, followed to delivery. The trait anxiety scale of the State-Trait Anxiety Inventories was used in order to detect a propensity towards anxiety. The association between anxiety propensity (defined as trait-anxiety subscale score above 38) and adverse pregnancy outcome was evaluated. Primary outcome was a composite outcome including preterm birth prior to 37 gestational weeks, hypertensive disorders in pregnancy, small for gestational age newborn and gestational diabetes mellitus. Secondary outcomes were each one of the above mentioned gestational complications.

**Results:**

There were no significant between-group differences in adverse pregnancy outcomes, including the rate of preterm birth, hypertensive disorders, small for gestational age, gestational diabetes or a composite outcome of them all.

**Conclusion:**

Anxiety propensity is not associated with adverse pregnancy outcome.

**Electronic supplementary material:**

The online version of this article (10.1186/s12884-018-1925-8) contains supplementary material, which is available to authorized users.

## Background

Psychological conditions during gestation and their impact on perinatal outcome is a matter of debate. One of the most prevalent conditions is anxiety, affecting approximately one-third of pregnant women, at some point during gestation. Anxiety could be dichotomized, with regards to pregnancy, according to its time of onset and etiology: 1) pregnancy-related anxiety (PRA), which is a subtype of state-anxiety as an anxiety responsive to a specific situation, i.e. pregnancy for this subtype. The main characteristic of this type of anxiety is excessive worry or fear of the unknown or of a specific event, such as pregnancy complications, labor conducts or fetal abnormalities; 2) pre-pregnancy anxiety, which exists as a part of the woman’s personality as a DSM (Diagnostic and Statistical Manual of Mental Disorders) defined disorder (e.g. Generalized Anxiety Disorder), or as anxiety-prone personality trait-anxiety or anxiety propensity [1–5]*.*

Prior reports of anxiety and its effect on pregnancy outcome have shown ambiguous and inconsistent depictions. Some studies report an association between different types of anxiety and a number of obstetrical and neonatal complications. Pregnancy related anxiety may increase the risk of preterm birth (PTB) [6–8], low birth weight (LBW) infants [9] and preeclampsia [10]. Pre-pregnancy anxiety, as a woman’s propensity, was positively associated with increased risk of LBW [11]*,* small for gestational age (SGA) [12], pregnancy related hypertensive disorders [13]_**,**_ PTB and cesarean delivery [14–17]. Other non-specific types of anxiety have been correlated to PTB [18, 19], SGA [20, 21] and LBW [22]. However, other studies have not found a significant association between anxiety during pregnancy [4, 22–24] or DSM-defined anxiety disorders [25, 26] to adverse pregnancy outcomes.

One of the possible pathophysiological mechanisms underlying anxiety association to adverse pregnancy outcome is based on the fact that an anxiety-prone emotional state is associated with continuous stress, a factor that was linked to a variety of adverse pregnancy outcomes [27–29]. Stress increases levels of corticotrophin-releasing hormone (CRH), which expressed abundantly in the placenta and in maternal and fetal plasma. It is thought to play a significant role in the regulation of fetal maturation, timing of delivery and fetal-placental blood flow. Elevated CRH concentrations, as compared with gestational age matched controls, are associated with preterm labor [30]. Nevertheless, the relationship between measurements of stress and pregnancy complications is not straightforward in large epidemiological studies [31]**.**

Another possible mechanism is based on inflammatory processes as it is well-established that anxiety symptoms predict dysregulation of inflammatory processes. Successful pregnancy has been associated with attenuated pro-inflammatory cytokine production in response immune challenges, while elevations in pro-inflammatory cytokines are causally implicated in preterm birth. Pro-inflammatory cytokines can promote preterm labor by triggering preterm contractions, encouraging cervical ripening and causing rupture of membranes [32].

Even though the effect of maternal stress on pregnancy outcome has been well studied, the role of anxiety as an independent risk factor for obstetrical and neonatal complications is less consistent.

These discrepancies, as reported in prior reports, may be due to inadequate control for the exact timing and type of anxiety. Evidence suggests that different types of anxiety, especially anxiety related to pregnancy versus general anxiety as a trait, demonstrate different associations with pregnancy outcomes [33, 34] and should be treated as distinct clinical entities [35]. Moreover, most of the current research deals with pregnancy related anxiety or anxiety symptoms detected during pregnancy. These symptoms were shown to vary over the course of pregnancy [36, 37]. There is paucity of data regarding a stable state of prenatal anxiety, which is the main feature in anxiety prone personality, and whether it is associated with adverse pregnancy outcome.

Given these inconsistent results and the need for further prospective evidence to obtain a better understanding of specific risk constellations for anxiety during pregnancy, we aimed to isolate maternal anxiety propensity, and evaluate its association with adverse pregnancy outcomes - PTB, SGA, gestational diabetes mellitus (GDM) and hypertensive disorders of pregnancy. As a general state, which is not related directly to pregnancy, anxiety propensity is a relatively stable state, easier to detect and therefore more practical in terms of pre-pregnancy and pregnancy management. This kind of association may carry important practical tools such as possible personality evaluation before and during pregnancy, in order to anticipate and treat possible outcomes.

## Methods

We conducted a prospective cohort observational study of women attending routine clinical care in our hospital, in order to determine the impact of maternal anxiety propensity on adverse pregnancy outcome.

The study was approved by the Institutional Review Board of the Rabin Medical Center (Approval No. 0561–13-RMC). Written informed consent was provided by all participating women.

### Study population

Study population was comprised of pregnant women who visited the labor and delivery unit, ultrasound unit, and maternal-fetal medicine unit at the Helen Schneider Hospital for Women of the Rabin Medical Center in Petah-Tikva, Israel, from April to November 2014.

Eligibility criteria included all pregnant women, with a singleton gestation, at any gestational age, older than 18 years. Exclusion criteria were: (1) History of any medically diagnosed mental or psychological disorders; (2) Language limitation preventing independent completion of questionnaires; (3) Use of psychotropic agents, mood stabilizers, anxiolytics or antidepressant medications before and/or during pregnancy and (4) any fetal genetic or structural malformations.

### Data collection

Women who agreed to take part in the study, after signing informed consent, were requested to fill out a self-designed questionnaire to acquire demographic information, including: age, level of education, occupation, religion, marital status, general medical history, habits, major life events and possible stress factors prior and during pregnancy, as well as data regarding physical activity, alcohol consumption and smoking. The second step of data collection was applying a standardized anxiety scale - the Trait Anxiety Scale (T-Anxiety) of the State-Trait Anxiety Inventory (STAI) [38]. The STAI is a standardized self-report questionnaire which is widely used to reliably measure the presence and severity of current symptoms of anxiety and the level of generalized anxiety propensity. The English version of the STAI questionnaire is depicted in appendix A (in the Additional file 1). We used a verified Hebrew translation, where question 21 through 40, corresponds to the T-anxiety scale [39, 40].

The STAI has separate subscales which measure these two types of anxiety, and clearly differentiates between them: The State Anxiety Scale (S-Anxiety) which evaluates the current state of anxiety, and The Trait Anxiety Scale (T-Anxiety) which evaluates stable aspects of anxiety proneness, defined as an acquired behavioral disposition that makes an individual susceptible to perceiving a wide range of objectively harmless situations as threatening and to react to them with the anxiety states. STAI has a significant high validity since its scores are positively correlated with other scales that measure anxiety and present high reliability [41, 42]. The STAI includes 40 items, 20 items allocated to each of the S-Anxiety and T-Anxiety subscales. Responses for the T-Anxiety scale assess frequency of feelings, as a general evaluation of their existence, in a 4-degree ruler: 1) almost never; 2) sometimes; 3) often; and 4) almost always. The range of scores for each subtest is the sum of responses, according to the ruler, for each of the 20 items in the questionnaire. Accordingly, the STAI T-Anxiety result can range from 20 to 80, where the higher score indicates greater level of anxiety. Score of 38 was chosen as a cutoff score to define proneness to anxiety, based on normative STAI results observed in similar populations as in our study [43]. In the statistical analysis, this cut off score was later turned out to also represent the 75th percentile in our population.

Data collection was continued until delivery to collect gestational complications occurring for each participant and pregnancy outcomes, including: mode of delivery, type of delivery onset, gestational age at delivery, birth weight and the occurrence of pregnancy complications such as GDM and hypertensive disorders.

### Outcome measures

Due to the relatively small incidence of each one of the pregnancy complications we referred, and our objective for sufficient statistical power, we chose the primary outcome to be a composite adverse pregnancy outcome including PTB prior to 37 gestational weeks, hypertensive disorders in pregnancy (gestational hypertension or preeclampsia), SGA and GDM. Secondary outcomes were each one of the above gestational complications.

### Definitions

Gestational age was calculated by the reported last menstrual period (LMP) and was adjusted to crown-rump length (CRL) assessment if a 7 day gap or greater was detected between LMP dating to 1st trimester CRL. Preterm delivery was defined as delivery prior to 37 completed weeks of gestation. SGA was defined as sex-customized actual birth weight percentile below the 10th percentile for a given gestational age, according to local growth curves [44]. The diagnosis of GDM required a 100 g 3 h oral glucose tolerance test, with any two of the four plasma glucose values to be equal or more than: fasting - 95 mg/dl, post glucose ingestion: 1 h - 180 mg/dl, 2 h - 155 mg/dl, 3 h - 140 mg/dl [45]. Hypertensive disorders were defined according to ACOG criteria [46].

### Statistical analysis

Data were analyzed using the software program SAS (Statistical Analysis System, SAS Institute Inc. Cary, North Carolina, USA), version 9.4. Continuous variables are expressed as the mean ± standard deviation (SD) and categorical variables as a numbers and percentage. Independent samples *t*-test and the χ^2^-test were used to compare continuous and categorical differences, respectively. A *p*-value of ≤.05 was considered statistically significant.

Giving the outcome’s incidence (GDM 7%, hypertensive disorders 5%, PTB 12%, SGA 10%) and considering overlapping, we expected the primary outcome to manifest in about 25% of the control group. For a predicted difference of 1.5 times in the study group, with 5% level of significance and 80% of power, the desired sample size according to Z-test for proportion comparison was 430 women.

## Results

The initial study population included 582 pregnant women at 24 to 40 gestational weeks, of which 64 were lost to follow up, without available birth outcomes. Another 6 participants did not fill out the STAI. Thus, data from a total of 512 women were available for analysis. Participants were divided into two groups using maternal T-Anxiety score. Women whose score was above 38, were considered ‘anxiety prone’ (*n* = 124, 24.2%) and the remainder of the cohort was considered to be ‘not anxiety prone’ (*n* = 388, 75.8%).

Baseline characteristics of the study population, stratified according to maternal anxiety propensity, are listed in Table 1. Anxiety prone women, compared to the non-anxious group, had significantly higher body mass index (*p* = .04) and were significantly less educated (*p* < .001). No significant differences were noted with other maternal parameters. Mean level of T-anxiety score in women in the second trimester of their pregnancy was 35.75 and in women in their third trimester was 32.93. This difference was not statistically significant.

**Table 1:** Study population characteristics, stratified according to maternal anxiety propensity

*p*-value	Anxiety prone (*n* = 124)	Not anxiety prone (*n* = 388)	Parameter
Maternal age, years	31.89 ± 4.81	32.06 ± 5.12	0.95
Body mass index, Kg/m^2^	23.67 ± 4.51	24.98 ± 5.83	0.04
Nulliparity	162 (41.75)	44 (35.48)	0.20
Number of living children	1.07 ± 1.15	1.07 ± 1.08	0.90
Use of assisted reproductive techniques	34 (8.92)	13 (10.92)	0.59
Mode of delivery:			
Vaginal Delivery	255 (65.72)	81 (65.32)	0.16
Cesarean Delivery	94 (24.23)	37 (29.84)	
Operative Vaginal Delivery	37 (9.54)	6 (4.84)	
Onset of Labor:			
Spontaneous	185 (47.68)	55 (44.35)	0.61
Augmentation	25 (6.44)	8 (6.45)	
Induction	106 (27.32)	31 (25.00)	
Elective Cesarean, no trial of labor	67 (17.27)	28 (22.58)	
Coffee consumption, cups per day^a^	1.83 ± 1.63	1.83 ± 1.46	0.74
Education, years	15.15 ± 2.45	14.43 ± 2.50	<.001
Physical activity	163 (42.56)	44 (36.67)	0.29
Drinking alcohol	51 (13.25)	14 (11.57)	0.76
Smoking^a^	76 (19.74)	32 (26.45)	0.13
Living place, Urban^a^	365 (84.66)	118 (83.90)	0.88
Place of birth, Local	365 (94.07)	118 (95.16)	0.06
Job loss^a^	9 (2.35)	4 (3.36)	0.52
Death of relative^a^	19 (4.97)	7 (5.79)	0.81
Moving house^a^	55 (14.36)	17 (14.05)	1.00
Work status, employed^a^	357 (93.46)	108 (90.76)	0.31
Gestational age at delivery, weeks	38.66 ± 2.31	38.23 ± 2.17	0.11
Birth weight, grams	3163.84 ± 607.33	3099.94 ± 587.01	0.29
Birth weight, percentile	54.07 ± 27.84	53.17 ± 27.37	0.73

Continuous variables are presented as median (range), Categorical values are presented as n(%)

^a^up to 2 months prior to pregnancy

There were no significant between-group differences in adverse pregnancy outcomes (Table 2).

**Table 2:** Adverse pregnancy outcome, stratified by maternal anxiety propensity

Outcome	Not anxiety prone *n* = 388	Anxiety prone *n* = 124	*p*-value
Preterm birth prior to 37 weeks	47 (12.11)	14 (11.29)	0.87
Hypertensive disorders in pregnancy	14 (3.67)	5 (4.17)	0.79
small for gestational age	23 (5.93)	10 (8.06)	0.40
Gestational diabetes mellitus	33 (8.66)	11 (9.17)	0.85
Composite outcome^a^	98 (25.59)	36 (29.75)	0.41

Data presented as n (%)

^a^Composite outcome includes any of: preterm birth prior to 37 gestational weeks, hypertensive disorders in pregnancy, small for gestational age, gestational diabetes mellitus

## Discussion

We conducted a prospective observational study aimed to evaluate the association between maternal anxiety propensity and adverse pregnancy outcome among 512 women. The results of the study suggest that women who have a tendency towards anxiety are not at an increased risk for adverse pregnancy outcome including PTB, SGA, GDM or hypertensive disorders.

Our results may be supported by findings from several other studies. Dayan et al. [24] assessed anxiety using the STAI and have not found a significant association between maternal trait anxiety and PTB among women with no history of preterm labor (OR = 0.92; 95% CI, 0.40–2.10). Qiao et al. [4] and Berle et al. [23] evaluated anxiety using the Hospital Anxiety and Depression Rating Scale (HADS) and found no significant association between anxiety symptoms during pregnancy with PTB, LBW, growth restriction or other adverse perinatal outcomes. The HADS is a screening measure tailored to detect the presence of symptoms of anxiety in medically ill patients and therefore is probably less suitable for healthy pregnant women. Moreover, it is designed to measure symptoms of anxiety and not anxiety as a trait [39]. A meta-analytic review by Littleton et al. [22] was conducted with data of 50 studies which assessed different types of anxiety. They found no significant associations between anxiety symptoms to PTB and other adverse perinatal outcomes. Finally, Maina et al. [25] and Andersson et al. [26] have not detected a significant association between DSM-defined anxiety disorders and adverse pregnancy outcome.

A recent Systematic Review and Meta-Analysis by Rose et al. [34] has reported a significant association between prenatal maternal anxiety and PTB. Although the main goal of this study was to appraise the effect of methodological heterogeneity on PTB, they emphasize that it is hard to distinguish the effect of the various types of anxiety on PTB. This important element gives more strength to the importance of differentiating anxiety types when analyzing their association with pregnancy outcome.

Most of the current available literature is focused on pregnancy related anxiety, but its detection and management have been limited by the scarcity of valid screening tools [27]. Therefore, anxiety propensity as a distinct and specific type of anxiety which is relatively stable, easier to detect and not related directly to pregnancy, may carry important practical clinical significance such as possible personality evaluation before and during pregnancy, in order to anticipate and treat possible outcomes.

Considering the different results derived while inspecting different types of anxiety and the lack of data regarding a stable state of anxiety, the isolation of maternal anxiety propensity is an important strength of our study. Another strong feature of our study is its prospective nature which more clearly indicates the temporal sequence between anxiety propensity and pregnancy outcomes as well as helps avoiding recall bias. Last, although based clinically on normative results in similar population as in our study, the cutoff score to define proneness to anxiety turned out to also represent the 75th percentile, thus strengthen the clinical validity.

A limitation of the study is the fact that the assessment of anxiety propensity was made during pregnancy, raising questions about the power of anxiety diagnosis and the specific type of anxiety detected. Nevertheless, the Trait Anxiety Scale (T-Anxiety) was used, which evaluates relatively stable aspects of anxiety proneness, attempting to eliminate the present state of anxiety as a confounder. Another drawback regarding assessment during pregnancy is the possible indefinite measurement-outcome chronological order in part of the cases, which may impair the prospective nature of the study. However, the measurement of trait anxiety minimizes this impairment as it intended to detect a baseline personality disposition independent on specific timing. Last, anxiety propensity can co-occur with other conditions such as depression and general stress, which poses them as possible confounders. Exclusion of all patients with history of mental or psychological disorders and all users of psychotropic agents, mood stabilizers, anxiolytics or antidepressant medications, as well as the use of a validated, reliable questionnaire designed specifically for anxiety detection - was targeted to minimize this possible bias. Nevertheless, further studies employing larger cohorts are needed, which will allow isolating a number of important pathologic pregnancy outcomes and studying the effects of maternal anxiety propensity on each one of them separately.

## Conclusion

In conclusion, this body of evidence, which parallel to a developing research regarding the possibility of mental disorders to be an important component of the risk profile for adverse pregnancy outcome, sets the stage for more collaborative psychological medical inter-disciplinary research, to inquire for anxiety propensity in pregnant women. It is critical to identify the signs, symptoms, and diagnostic thresholds that warrant prenatal intervention and to aspire developing efficient and valid screening and intervention strategies.

## Additional file

Additional file 1: Appendix A. Standardized anxiety self-report questionnaire to assess the Trait Anxiety Scale of the State-Trait Anxiety Inventory. (PDF 150 kb)

## Abbreviations

CI: Confidence interval
CRH: Corticotrophin-releasing hormone
CRL: Crown-rump length
DSM: Diagnostic and Statistical Manual of Mental Disorders
GDM: Gestational diabetes mellitus
HADS: Hospital Anxiety and Depression Rating Scale
LBW: Low birth weight
LMP: Last menstrual period
OR: Odds ratio
PRA: Pregnancy-related anxiety
PTB: Preterm birth
S-Anxiety: The State Anxiety Scale
SAS: Statistical Analysis System
SD: Standard deviation
SGA: Small for gestational age
STAI: State-Trait Anxiety Inventory
T-Anxiety: The Trait Anxiety Scale
